# Serous Papillary Adenocarcinoma of Unknown Primary in a Recurrent Paravaginal Cyst

**DOI:** 10.1155/2019/8125129

**Published:** 2019-06-10

**Authors:** Khilen Patel, Advaita Punjala-Patel, Angela Stephens, John Lue

**Affiliations:** Department of Obstetrics and Gynecology, Augusta University, Medical College of Georgia, 1120 15th St., Augusta, GA 30912, USA

## Abstract

Cystic lesions located in the paravaginal region are rare. When present, paravaginal cysts are typically benign and are incidentally found on routine gynecological exams; however, rarely they can be malignant. Treatment options for paravaginal cancers are not well studied and early diagnosis may help improve prognosis in these patients. Our case describes a 55-year-old female with a recurrent paravaginal cyst that was remarkable for serous papillary adenocarcinoma despite biopsy and fluid cytology negative for malignancy. This case demonstrates that malignancy should be considered highly with a recurrent paravaginal cyst, especially when present over a short interval.

## 1. Introduction

Large paravaginal cysts are rare and when present are most commonly benign. These cysts can be either congenital or acquired. While congenital cysts are associated with developmental abnormalities and are typically derived from embryological remnants, acquired cysts are most commonly caused by trauma [1]. There have been only a few reported cases of malignancy found in paravaginal cysts; therefore, the typical management of paravaginal cysts has been primary vaginal drainage. Adenocarcinoma is a common malignancy that is prevalent in the endometrium, ovary, and vagina among other gynecological sites. Serous papillary adenocarcinoma (SPAC) is an aggressive subtype with a poor prognosis [2, 3]. Here, we present the case of a patient with a recurrent paravaginal cyst that was negative for malignancy on multiple aspirations, but on further investigation via laparotomy was remarkable for serous papillary adenocarcinoma.

## 2. Case Report

A 55-year-old postmenopausal female, gravida 0, presented for an annual gynecologic exam. Her surgical history was significant for a total abdominal hysterectomy with bilateral salpingo-opherectomy in 1986 for severe endometriosis. She had been taking conjugated estrogen daily since the operation due to postmenopausal symptoms and denied any vaginal bleeding or vaginal discharge. On bimanual examination, the uterus and cervix were noted to be surgically absent; however, a large pelvic mass was palpated. This mass was smooth, approximately 8 cm in diameter, and located at the apex of the vaginal vault. It was palpable rectally with no evidence of generalized lymphadenopathy. Stool guaiac test was negative.

**Figure 1:**
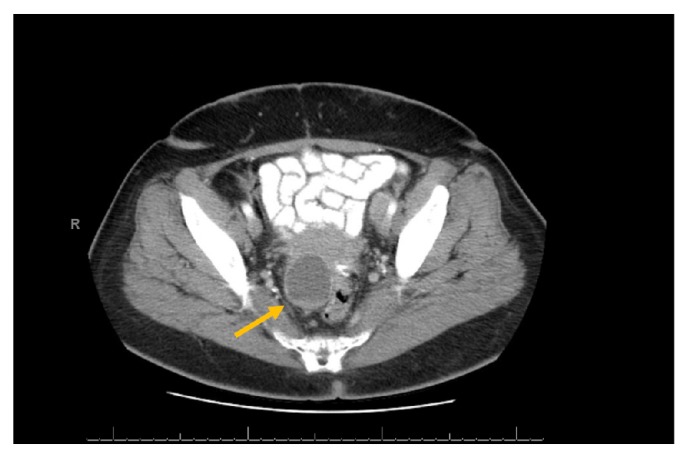
Sagittal image from CT scan following the intravenous administration of 100mL of Omnipaque 350 and 32 ounces oral Omnipaque 350 demonstrating simple paravaginal cystic structure with fluid density contents.

Diagnostic tests to further investigate the mass included vaginal cuff cytology, imaging, and laboratory studies. A Papanicolaou test was benign. Serum Cancer Antigen-125 was normal at 8.4 U/mL. Computed tomography of the abdomen and pelvis (CT) revealed a 10 cm mass posterior to the bladder, compressing the rectum to the left (Figure 1). The central portion of the mass was predominantly cystic with increased attenuation of the peripheral soft tissue border on the right lateral aspect. A diagnostic laparotomy was subsequently performed. The paravaginal cyst was drained, and the cyst wall was biopsied. Final pathology was benign.

**Figure 2:**
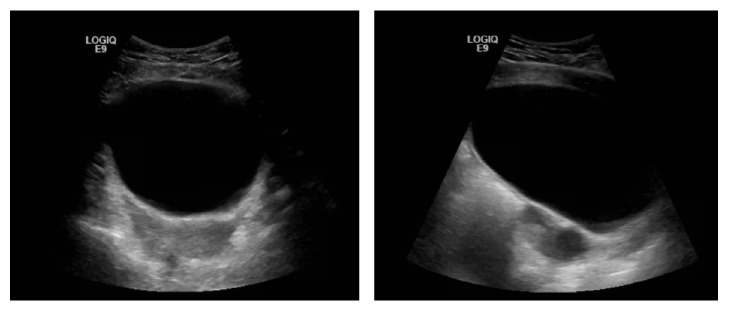
Abdominal ultrasound performed prior to cyst aspiration demonstrating recurrent, simple paravaginal cyst.

Approximately two weeks later, the patient presented to clinic with right upper and lower quadrant pain, vaginal spotting, and watery vaginal discharge. There was no rebound tenderness or guarding present. A pelvic exam was unremarkable; the vaginal cuff was intact with no evidence of any masses or lesions. CT was notable for a right sided cystic mass that displaced the bladder and rectosigmoid colon to the left. Ultrasound-guided aspiration of the 7.67 x 7.76 cm loculated cystic mass was performed revealing 150 cc of serous fluid, with immediate relief in the patient's symptoms (Figure 2). Due to clinical findings suggestive of a possible pelvic abscess, the patient was placed on antibiotics; however, the final aerobic and anaerobic cultures from the cystic fluid were negative for any infectious or malignant process.

Three weeks following aspiration of the cyst, the patient returned to clinic with complaints of right pelvic pain. Transvaginal ultrasound showed a recurrence of the 7 cm cyst, which was aspirated to reveal 270 cc of serosanguinous fluid. Cytology of the fluid was negative for malignancy and consistent with a benign cyst. Interestingly enough, a third recurrence of the cyst was noted with 230 cc of subsequent serous fluid drainage. Given the multiple recurrences and unpredictable nature of cyst, a laparotomy and right cystectomy was scheduled.

Surgical findings were significant for a large fluctuant mass in the right pelvis extending to the posterior peritoneum. The posterior-lateral capsular wall of the cyst was noted to be nodular and extensively adherent to the posterior peritoneum and pelvic side wall. Because of the extent of the adhesions, a combined transvaginal and abdominal resection was necessary. The right vaginal wall was subsequently entered, and the cyst wall was dissected from the deep pelvic side wall. Intraoperative frozen section of the cyst was notable for serous papillary adenocarcinoma. At this point in time, Gynecology Oncology was consulted intraoperatively and an omentectomy and peritoneal washings were performed which were also negative for malignancy. A thorough exploration of the pelvic cavity was only notable for a small cystic mass at the top of the sigmoid colon, which was excised and found to be a benign serous cyst.

Final pathology of the paravaginal cyst wall was remarkable for a benign squamous lining with scattered cell fragments of degenerated papillary configuration consistent with serous papillary carcinoma. The nodules on the posterior wall of the cyst showed necrotic stroma with dystrophic calcification representing infiltration of moderate to poorly differentiated serous papillary carcinoma of unknown primary. Further review of tumor markers demonstrated that the wall of the cyst was estrogen receptor (ER) and progesterone receptor (PR) negative; however, it was positive for Herceptin, CA-125, and focally positive for Wilms Tumor (WT-1), consistent with serous papillary carcinoma (Table 1).

Preoperative imaging of the patient was unremarkable for metastasis or any suspicious lesions. Given the findings, a postoperative head CT was performed which revealed no evidence of intracranial metastasis. The patient was subsequently referred to Gynecology Oncology where thorough discussions were held regarding palliative therapy versus chemotherapy and radiation. She was counseled on the limited data currently available on the treatment of vaginal cancer, and all acute and long-term serious sequelae, along with her poor prognosis, were further explained to the family. After many discussions, she was planned to start on once weekly cis-platinum chemotherapy given intravenously at a dose of 50 mg/m^2^ with concurrent external beam radiation plus interstitial brachytherapy. After receiving three courses of her chemotherapy, the patient refused additional treatment due to inability to tolerate the side-effects. Despite undergoing external beam radiation plus interstitial brachytherapy for three months, the patient experienced rapid clinical deterioration with subsequent vesicovaginal fistula formation, and the decision was made for palliative therapy and pain management after three months of aggressive treatment.

## 3. Discussion

Paravaginal cysts are asymptomatic and are incidental masses that are usually found during routine gynecological exams [4]. These cysts are estimated to be present in approximately 1 in 200 women and are most commonly found in the third and fourth decades. Most are congenital, arising from embryological remnants, while others are acquired resulting from trauma [1]. Classification of cysts is generally based on histologic and histochemical features of cyst epithelium. Congenital cysts are typically benign and are further classified by embryologic structures—Mullerian duct, mesonephric ducts (Gartner's cyst), or urogenital sinus (Skene's cyst) [5]. Primary carcinomas of the vagina originating from these cysts account for only 1 to 2% of gynecological cancers and are very rare. Thus, the primary management of these cysts includes drainage and excision. Squamous cell carcinoma is the most common type of malignant transformation, but adenocarcinoma can occur as well. Adenocarcinoma is a common gynecological malignancy that is present in postmenopausal females, and an aggressive subtype of this cancer is serous papillary cancer. Many vaginal adenocarcinomas typically occur in young adolescents exposed to Diethylstilbestrol (DES) and have a good prognosis; however, the natural disease progression of those who present without DES exposure is grave [6]. Treatment of these cancers is typically multimodal and involves both radiation and chemotherapy [7]. Multiple imaging modalities, including an MRI and transvaginal ultrasound, should be utilized when identifying a paravaginal cyst, and surgical excision via laparoscopy or laparotomy in patients with recurrent paravaginal cysts should be performed to further investigate gynecological malignancy.

Currently, treatment options and studies existing for the treatment of paravaginal cancers are extremely limited. The combination of external beam radiation and chemotherapy has been studied extensively by numerous investigators [7–9]. McCurdy and Zouain (2009) described the successful treatment of vaginal papillary serous adenocarcinoma in a patient with DES exposure with combination of external beam radiation and cis-platinum [7]. Samant et al. investigated 28 patients with primary vaginal adenocarcinoma and described the use of weekly cis-platinum therapy with combined external beam radiation and its positive impact on overall survival [9]. High dose radiation (HDR) brachytherapy has been shown to decrease the incidence of vaginal cuff recurrence in papillary serous carcinoma of the uterus and has been implicated in increasing survival in patients with uterine clear cell carcinoma [10]. Given that the patient had papillary serous carcinoma of an unknown primary, HDR brachytherapy with concurrent whole pelvic radiation alone would have been a good alternative in management; however, cis-platinum was used as an adjuvant in this patient given her history of recurrence and to sensitize the tumor bed [7, 11]. Though interstitial brachytherapy is generally well tolerated, side-effects include proctitis, cystitis, and, more severely, rectovaginal fistula formation requiring colostomy [12].

This case demonstrates a serous papillary carcinoma that was not found on primary aspiration of a paravaginal cyst, but was found in the cyst wall once it was excised after three subsequent recurrences. It illustrates that malignancy should be considered highly on the differential diagnosis with a recurrent paravaginal cyst, especially when they persist within a short interval. Perhaps, if it had been discovered earlier, the patient would have had a better prognosis with concurrent radiation and chemotherapy, but given the multiple recurrences and the delay in time to treatment, the patient in this case deteriorated rapidly.

## Figures and Tables

**Table 1:** Pertinent tumor marker results of recurrent paravaginal cyst wall.

Tumor Marker	
CA-125	Positive
Estrogen Receptor	Negative
Herceptin Receptor	Positive
p53	Unknown
Progesterone Receptor	Negative
Wilms Tumor (WT-1)	Focally positive
